# Analysis of Occupational Accidents in Underground and Surface Mining in Spain Using Data-Mining Techniques

**DOI:** 10.3390/ijerph15030462

**Published:** 2018-03-07

**Authors:** Lluís Sanmiquel, Marc Bascompta, Josep M. Rossell, Hernán Francisco Anticoi, Eduard Guash

**Affiliations:** 1 ICL Chair in Sustainable Mining, Polytechnic University of Catalonia, 08034 Barcelona, Spain; marc.bascompta@upc.edu; 2 Department of Mathematics, Polytechnic University of Catalonia, 08034 Barcelona, Spain; josep.maria.rossell@upc.edu.; 3 Department of Mining Engineering, Industrial and ICT, Polytechnic University of Catalonia, 08034 Barcelona, Spain; hernan.anticoi@upc.edu (H.F.A.); eduard.guasch@upc.edu (E.G.)

**Keywords:** data mining, association rules, previous cause, type of accident, overexertion

## Abstract

An analysis of occupational accidents in the mining sector was conducted using the data from the Spanish Ministry of Employment and Social Safety between 2005 and 2015, and data-mining techniques were applied. Data was processed with the software Weka. Two scenarios were chosen from the accidents database: surface and underground mining. The most important variables involved in occupational accidents and their association rules were determined. These rules are composed of several predictor variables that cause accidents, defining its characteristics and context. This study exposes the 20 most important association rules in the sector—either surface or underground mining—based on the statistical confidence levels of each rule as obtained by Weka. The outcomes display the most typical immediate causes, along with the percentage of accidents with a basis in each association rule. The most important immediate cause is body movement with physical effort or overexertion, and the type of accident is physical effort or overexertion. On the other hand, the second most important immediate cause and type of accident are different between the two scenarios. Data-mining techniques were chosen as a useful tool to find out the root cause of the accidents.

## 1. Introduction

The mining industry is an important economic sector in many countries, including Spain, and includes coal, metal, and non-metal minerals. These minerals are used extensively worldwide and are required for the production of necessary elements in all sectors, such us construction, energy, agriculture, medical and electronic. According to the mining statistics of the Ministry of Industry, Energy and Digital Agenda of Spain, in 2015, there were 2896 surface and underground mines, employing 4540 people in underground mines, 12,702 in surface mines and 6755 in mineral processing plants.

Many studies have pointed out that the mining sector is one of the most dangerous due to its intrinsic characteristics: humidity, dust exposure, danger of injury from falling rocks, falls on the same and different level, etc. [1,2,3,4,5,6,7]. All these factors have an influence on the incidence and severity of accidents. According to the Spanish administration data, the incidence rate of accidents per 100,000 workers was 4.3 times higher in the mining sector than the overall incidence among all other sectors in 2015, confirming the danger of mining compared to other economic sectors. Furthermore, the incidence rate of accidents among underground miners was 4.8 times higher than in surface mining between 2005 and 2015. Comparing this accident rate with the mining sector of other countries, the Spanish ratio is 5.5 times higher than in the United States and 16.1 higher than in Australia, according to the data from the National Institute for Occupational Safety and Health (NIOSH) and the Department of Natural Resources and Mines (DNRM) of Queensland. However, the Spanish incidence rate has been significantly reduced; by 2.1 times in the period 2005–2015. Despite this remarkable reduction, there is still room for improvement, which would reduce the direct and indirect costs generated by injuries and deaths [8]. Many studies related to high-risk industries have shown that safety culture and organizational performance are key factors in reducing workplace accident rates [9,10,11,12,13].

The accident analysis in Spanish surface mining between 2003 and 2012 showed that one of the main causes of accidents was physical overexertion on the musculoskeletal system, with body movement being the most common immediate cause [14]. Moreover, some studies have also indicated a direct relationship between overexertion injuries and the age of the employee [15].

Safety management decisions that must be made to select and prioritize problem areas and safety system weaknesses must be based on the recognition of hazards encountered in each activity of the mining process [16]. For this reason, knowledge of the major occupational hazards, their causes and factors, is necessary in order to improve the safety conditions of the employees. Occupational injury/accident datasets are often large and complex and require a tool able to handle large amounts of information for analysis. One such approach is data mining, which can examine the genesis of occupational accidents, together with the extraction of rules or behavioral patterns of injuries.

Methods based on Bayesian networks are more sensitive in detecting associations among categorical variables than other statistics methods [17,18]. Hence, this methodology can be very useful for obtaining reliable conclusions for the decision-making process with regard to safety issues. Bayesian networks have also been applied in many other scientific fields, including civil engineering [19], geological engineering [20], ecology [21], medicine [22], road traffic safety [23,24,25], environmental assessment impact [26,27], business risk and product life-cycle analysis [28], workplace tasks [29], workplace risk areas [30], and the interrelation between hygienic workplace conditions and occupational accidents [31] or construction and mining accidents [32,33].

The objective of this study was to extract information from the annual digital database of the Spanish Ministry of Employment and Social Safety between 2005 and 2015 and to identify the factors most strongly associated with accidents and injuries in this population. We identified the main factors influencing the typology of occupational accidents with the idea of improving prevention policies, minimizing risks, and reducing injuries and deaths in the mining sector.

## 2. Materials and Methods

### 2.1. Study Population

A total of 56,034 mining accidents were classified as either occurring in underground mines (Scenario I; *n* = 28,894) or surface mines (Scenario II; *n* = 27,140). Data was obtained from the Spanish Ministry of Employment and Social Safety between 2005 and 2015.

### 2.2. Variables

Although 54 variables were registered for every accident, we chose only 16 of them due to their relevance to the study. Subsequently, they were evaluated by data-mining software, which determined only 11 variables as relevant. The variable “Type of Accident” was selected as the target variable, while the remaining variables were considered predictors.

We use numerical and graphic statistical methods to identify errors in the data base and verify if they occur more within certain groups in the data. If so, the instances are corrected or removed from the original dataset. The 11 selected variables are the following:

1. Age (A): Age of the injured worker, distributed into seven classes: (16–24), (25–29), (30–34), (35–39), (40–44), (45–54), (≥55).
2. Contract (C): Employment contracts were classified as 1 = permanent and full-time; 2 = permanent and part-time; 3 = temporary and full-time; 4 = temporary and part-time.
3. Day Hour (DH): The hour of the day at which the accident occurred was classified as (0–6), (6–10), (10–14), (14–18], (18–24).
4. Experience (E): The prior work experience (in months) of the injured worker was classified into seven groups (0–12), (13–30), (31–60), (61–120), (121–180), (181–240), (≥241).
5. Physical Activity (PA): The physical activity done by the worker was classified as 1 = machine operations; 2 = working with hand tools; 3 = driving or being in a conveyance; 4 = manipulation of objects; 5 = manual handling of loads; 6 = performing a movement; 7 = others.
6. Place (P): Location at which the injury occurred was classified as 1 = treatment plants, workshops and storages; 2 = general constructions or demolitions; 3 = surface mine; 4 = underground mine; and 5 = other places. This variable was only considered in surface mining.
7. Preventive Organization (PO): The preventive organization system implemented by the company was classified as 1 = the employer; 2 = designated workers; 3 = own prevention service; 4 = joint prevention service; 5 = external prevention service; 6 = without prevention service.
8. Previous Cause (PC): Previous cause of the accident, grouped in seven categories: 1 = electric problem, explosion, fire, overflow, overturn, leak, spill, vaporization or emanation; 2 = fracture, slip, fall or collapse; 3 = loss of control of the working machinery, total or partial; 4 = falls/tumbles of a person; 5 = body movement without physical effort; 6 = body movement with physical effort or overexertion; 7 = other causes.
9. Size (S): Number of employees in the mine was grouped as (0–9), (10–19), (20–49), (50–99), (100–499), (≥500).
10. Type of Accident (TA): This variable explains the mechanism of the accident. Seven categories were considered: 1 = electric contact, fire, contact with hazardous substances, drowning; 2 = impact or collision with stationary object; 3 = hit or collision with a moving object; 4 = contact with a sharp or pointed object; 5 = being trapped, crushed or suffering an amputation; 6 = physical effort or overexertion; 7 = others.
11. Work Hour (WH): The number of h the employee worked before the accident occurred was grouped into six categories (0–1), (2–4), (5–8), (9–10), (11–12), (≥13).

*Remark:* We note that the intervals in which some variables are classified appear unequally distributed. The reason for this classification is that the information available from the database is supplied in this way.

**Figure 1:**
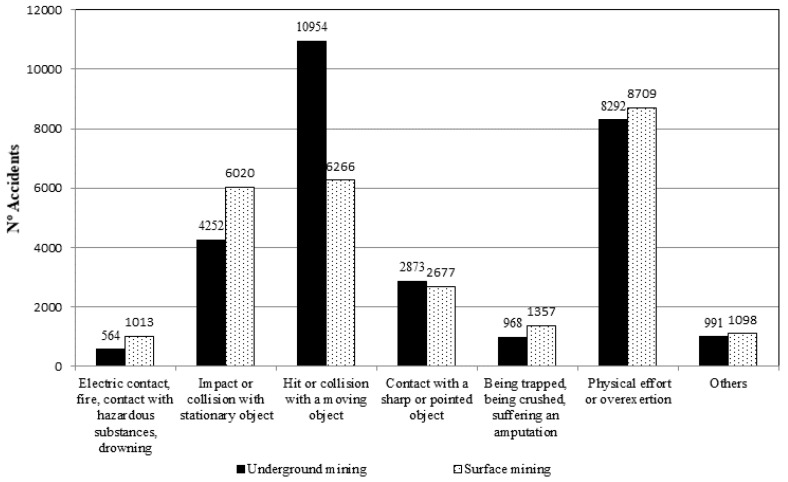
Frequency distribution for the target variable Type of Accident.

The parameters we created had a terminology that included the variable and the corresponding category, such as A1, which meant the age (A) of the injured worker was between 16 and 24 (1). Figure 1 shows the absolute frequency distribution for the variable Type of Accident, used as the target variable.

### 2.3. Selection of Predictor Variables

The objective of this subsection is to select the most significant variables that are relevant to making predictions. Feature selection is divided into two parts: Attribute Evaluator and Search Method, with multiple techniques to be chosen in each part. Attribute evaluator is a technique by which each attribute is evaluated in the context of the target variable. The search method is a technique by which to try or navigate different combinations of variables in the dataset to arrive on a short list of chosen features.

Weka 3.9 was used in this study. It is data-mining software that applies algorithms for data preprocessing, classification and regression, including Bayesian classifiers, decision trees, rule sets, linear regression and nearest-neighbor methods [34,35,36]. It provides methods for identifying the variables that are predictive for Type of Accident. In this study, some attribute evaluators, along with search methods, are applied (Table 1). Two different selection modes—called the full training set and cross-validation—are used. The dataset is randomly reordered and split into 10 folds of approximately equal size. A fold is used to test, while the others train the classifier.

We calculated the position of the variables in each learning scheme to determine the final ranking for the best predictors that influenced Type of Accident. Table 2 and Table 3 show the ranking of the predictor variables for Scenarios I and II, respectively. All the accidents in Scenario I belong to the same mine type, *p* = 4 (underground mine).

### 2.4. Classification Trees and Association Rules

The concept of the classification tree is similar to that of the regression model, i.e., a training set is used to create the model. This considers a database with known output values, which is used to construct the model and, if a new data instance with an unknown output value is put into the model, an expected output value is obtained. This is all the same as in the regression model. However, the methodology proposed in this paper takes it one step further: About 70–90 percent of the data are used for the training set, which will be utilized to create the model, and the remaining data are used to test its accuracy. For this, the confusion matrix shows how many instances have been correctly or incorrectly assigned to each class. Basically, a false positive is a data instance where the model predicts it should be positive, but the actual value is negative. Conversely, a false negative is a data instance where the model predicts it should be negative, but the actual value is positive.

There are many decision-tree algorithms, but one of the most common is the C4.5, a method of classification that generates a decision tree using the training data [37]. In this paper, an improved algorithm, the J48 implemented in Weka, has been used for Type of Accident as the target variable. When the J48 algorithm is applied, the confusion matrix is obtained. 77.4% of cases were properly assigned for Scenario I, and 70.6% for Scenario II, which is considered acceptable.

Association rules are obtained to identify relationships between attributes in data, working with the Predictive Apriori algorithm, which iteratively reduces the minimum support until it finds the required number of rules with the minimum confidence [38]. The confidence or predictive accuracy indicates the number of instances for which all the conditions are true, coverage, divided by the number of instances for which the conditions in the antecedent are true. Then, the best rules for Type of Accident allow extracting information about the main causes of the injuries for both scenarios.

## 3. Results

We obtained 130 and 110 association rules for Scenario I and Scenario II, respectively. The 20 best association rules for both scenarios are detailed in Table 4 and Table 5, sorted by their confidence level, with 3 or more predictor variables, and with the Type of Accident (TA) as the target variable, as well as the percentage of accidents related to the predictors and type of accident. Another ranking was also created, including the predictor variables ordered by the times one variable appears in the corresponding rule (Table 6 and Table 7). The number of associations was chosen with the idea of having an important number of them, while always remaining within an acceptable confidence range.

There are some important differences between the ranking from Weka (Table 2 and Table 3) and the number of times a variable appears in association rules (Table 6 and Table 7). Variables involved in more than 45% of all accidents are classified in lower positions by Weka. For instance, Contract (C), Preventive Organization (PO) and Work Hour (WH) have low positions in Scenario I according to the software classification, but they appear in most association rules. Additionally, C1, PO3 and WH2 are 82.7%, 74.9% and 51.5% of 28,894 accidents, respectively. Something similar happens in Scenario II, where the same variables are 83.2%, 62.2% and 48.4%, respectively, of 27,140 accidents.

Based on the data processed, the mining sector characteristics explain some of these initial outcomes. There is a high percentage (74.1%) of full-time and permanent work contracts, C1. Regarding the Prevention Organization system, internal prevention services were more common in underground mines (PO3), whereas external prevention services were more common in surface operations (PO5). Variable Work Hour (WH) has little influence on the Type of Accident. It only appears in 6th and 5th position in the top 20 for Scenarios I and II, respectively. Additionally, it was found that the majority of accidents occur between the first 2–4 working hours in 8 out of 11 rules in both scenarios, WH2.

### 3.1. Scenario I—Underground Mines

Previous Cause (PC), Size (S) and Physical Activity (PA) are on the top of the ranking of predictor variables according to Weka. Hence, these predictors are the most influential variables in the Type of Accident. 

PC is in 19 out of 20 association rules and the type PC6—body movement with physical effort or overexertion—is in 12 out of 19 rules, generating a TA6 accident: physical effort or overexertion. Meanwhile PC2—fracture, slip, fall or collapse—is in the other 7 rules, with a TA3 accident—hit or collision with a moving object—which is in accordance with previous studies [16,39]. According to these rules, accidents with a prediction variable PC6 and an outcome of TA6 are given in 91.6% of cases; whereas 39.9% have the prediction variable PC2 and an outcome TA3. 

The Size (S) variable, as measured by the number of employees, appears in the best 8 out of 10 association rules, with a class S5 in all cases, mining activities between 100–499 employees, and is a predictor variable in 57.6% of the accidents. The percentage of accidents, total workforce, and risk index for each mine size is shown in Table 8. The risk index is indicative of the incidence of accidents among different groups or subpopulations, and is defined as the ratio of percentage of injured workers of a given subpopulation to the percentage of the total workforce represented by this subpopulation. A risk index = 1.0 corresponds to an average risk, while a value greater than 1.0 indicates a higher risk for the group [40].

The risk index is higher in underground activities with fewer than 50 employees, compared to those with more employees, and it decreases as the mine size increases. This fact matches with other studies, both in the mining sector [41], and in other industrial activities [42]. Additionally, the difference between companies with a workforce of fewer than and more than 500 employees is quite relevant, as the latter case has a much lower risk index.

According to Weka, the Physical Activity (PA) developed at the moment of the accident is classified as the third-most influential variable in Scenario I, appearing in the best 9 out of 20 association rules (Table 4). The predominant activities are: manipulation of objects (PA4), and working with hand tools (PA2). On the other hand, Experience (E) is 4th in the ranking, with workers between 61 and 120 months of experience (E4) being the most important class. In reference to Age (A), it does not have a significant influence on the Type of Accident, being in the 7th and 8th positions in Table 2 and Table 6, respectively.

The three best association rules are as follows: Accident occurring while performing a physical activity manipulating objects with an immediate cause resulting from body movement with physical effort or overexertion is most common among:

1. Full-time permanent contract in a company with an internal prevention service.
2. Company with an internal prevention service and a size between 100 and 499 workers.
3. Full-time permanent contract in a company with an internal prevention service and a size between 100 and 499 workers.

### 3.2. Scenario II

Previous Cause (PC), Physical Activity (PA), Experience (E) and Size (S) are among the most important predictor variables, like in Scenario I, as well as the Place (P) variable, which is specific to surface mining and has the third-highest influence. On the other hand, variables Age (A), Preventive Organization (PO), Day Hour (DH), Contract (C) and Work Hour (WH) are at the bottom of the ranking, but A and DH have more relevance than in Scenario I.

Previous Cause (PC) appears in 18 out of 20 association rules and is of the class PC6 in 16 out of these 18—body movement with physical effort or overexertion—with a Type of Accident TA6; whereas falls/tumbles of a person (PC4) account for the other two, with impact or collision with stationary object (TA2), generating 114.4% and 11.9% of the accidents, respectively.

Place (P) is the third-most influential variable, and it appears in 7 out of 20 association rules (Table 5), with 6 of these rules being P1—treatment plants, workshops and storage—which includes 40.5% of all accidents. Regarding Experience (E), it is the 4th-most influential for accident generation. It appears in 3 out of 20, and in 23.7% of all accidents, as an association rule with the class E1—fewer than 12 months of experience.

The three best association rules are as follows: Accident occurring while performing a physical activity manipulating objects with an immediate cause based on body movement with physical effort or overexertion is most common among:

1. Full-time permanent contract and between the first 2–4 working hours.
2. Full-time permanent contract in a company with an external prevention service and between the first 2–4 working hours.
3. Less than 1 year of experience working in a treatment plant, workshop or storage.

## 4. Discussion

We identified the main factors influencing the typology of accidents using the software Weka and the database from the Spanish Ministry of Employment and Social Safety between 2005 and 2015.

However, we came across some limitations, such as determining whether an accident related to body movement with physical effort was produced due to human error or problems with the preventive organization. Additionally, when a few classes from a variable represent the majority of the cases the software reduce their importance. This pattern is found in Contract (C), Preventive Organization (PO) and Work Hour (WH). The fact that the majority of employees have a permanent contract, and that mines have the same type of preventive organization, does not mean that it is more likely to suffer an accident. Therefore, it is very important to know the weight of each class and the type of variable from the database for this type of analysis.

On the other hand, other variables show some clear evidence about the period of the day with the highest incidence, and this is reflected in the percentage of accidents. For example, the Work Hour (WH) with the highest probability of suffering an accident—within the first 2–4 h—suggests that there is a lack of attention due to a meal break, which is usual in this time slot, thus decreasing the concentration level when work is restarted [43]. With regard to the most Typical Accident (TA), whether for surface or underground mining, the immediate cause is body movement with physical effort or overexertion and it is related to the conditions in mines, which often require the use of tools in awkward positions, increasing the risk of injury [39].

In the case of underground mining, results show that preventive policies should be focused on reducing accidents due to physical effort or overexertion, perhaps mechanizing some actions, as well as increasing load handling training programs. In addition, falling of objects from the ceiling and walls is also an important Previous Cause (PC), as it is necessary to train workers about workplace inspections and control procedures. These statements are in accordance with [44], which studied 320 underground mines and concluded that the three most influential factors in coal mine accidents were: (1) lack of safety education and training; (2) rules and regulations for safety production responsibility; and (3) rules and regulations for supervision and inspection. Furthermore, Experience (E) displays a different behavior from that described in previous studies [14,42], where it was pointed out that the highest accident incidence was in cases with fewer than 30 months of experience. More research should be done to clarify these results.

Regarding surface mining, one of the most important variables is Place (P), which is in accordance with previous studies [13], confirming that treatment plants, workshops and storage are the workplaces with the highest incidence rate in surface mining. In the same direction, Experience (E) displays similar results to the previous literature [14,45]. However, this is in disagreement with results from Scenario I. Further research should be done to give insight into this apparent inconsistency. Preventive measures similar to Scenario I should be taken for the Type of Accident PC6-TA6 and PC4-TA2, due to their important consequences.

## 5. Conclusions

We demonstrated that data-mining techniques can be powerful tools for identifying the main factors affecting occupation accidents in the mining sector from a large database. The usage of Weka could help public and private companies to find out the root of the accidents, apply corrective measures and verify their effectiveness in time. We found some common patterns and differences between major predictors in underground and surface mining operations.

Among the five most influential variables, four of them were coincident; Previous Cause (PC), Size (S), Physical Activity (PA) and Experience (E). The most typical accident has an immediate cause of body movement with physical effort or overexertion, whether in surface or underground mining, while the second-most important type of accident varies between Scenarios I and II. An immediate cause of fracture, slip, fall or collapse (PC2) causing a type of accident of hit or collision with a moving object (TA3) is the most common in Scenario I. On the other hand, an immediate cause of falls/tumbles of a person (PC4) and a type of accident of impact or collision with stationary object (TA2) is the most usual in Scenario II. The Age (A) of injured workers has little influence on the type of accident, whereas the Size (S) of the company has more influence in the origin of the accident in Scenario I than in Scenario II.

## Figures and Tables

**Table 1:** Feature selection: Attribute evaluators, search methods and selection modes.

Target Attribute	Attribute Evaluators	Search Methods	Selection Modes
**Type of Accident**	ChiSquaredAttributeEval	Ranker	Full training set and 10 cross-validation
	CfsSubsetEval	GreedyStepwise ExhaustiveSearch BestFirst	
	ClassifierSubsetEval	RandomSearch	
	InfoGainAttributeEval	Ranker	

**Table 2:** Ranking of the variables for Scenario I.

Variables	PC	S	PA	E	PO	C	A	WH	DH
**Ranking**	1	2	3	4	5	6	7	8	9

**Table 3:** Ranking of the variables for Scenario II.

Variables	PC	PA	P	E	S	A	PO	DH	C	WH
**Ranking**	1	2	3	4	5	6	7	8	9	10

**Table 4:** The 20 best association rules for Scenario I with their corresponding confidence level and percentage of accidents for each association.

Predictor Variable 1	Predictor Variable 2	Predictor Variable 3	Predictor Variable 4	Target Variable	Confidence	% Accidents
C1	PC6	PA4	PO3	TA6	0.868	6.21
PC6	PA4	PO3	S5	TA6	0.867	6.30
C1	PO3	PA4	S5	TA6	0.863	7.10
E4	C1	PC6		TA6	0.842	5.05
E4	C1	PC6		TA6	0.828	5.60
C1	PC6	WH2	E4	TA6	0.816	8.37
C1	PC6	PO3		TA6	0.814	13.40
PC6	WH2	PO3		TA6	0.814	7.32
C1	PC6	WH2	PO3	TA6	0.814	7.20
S5	PC2	PA2		TA3	0.812	5.88
C1	PC2	PA4		TA3	0.811	6.52
S5	C1	PC2	PA1	TA3	0.804	5.34
PC2	PA2	WH2		TA3	0.803	5.73
PC2	PA4	PO3		TA3	0.799	5.73
C1	PC6	WH3		TA6	0.799	5.28
C1	PC2	PA4	PO3	TA3	0.799	5.59
S5	PC2	WH3		TA3	0.798	5.13
S5	C1	PC6	PO3	TA6	0.798	8.56
S5	PC6	PO3		TA6	0.797	8.72
S5	C1	PC6		TA6	0.796	9.59

**Table 5:** The 20 best association rules for Scenario II with their corresponding confidence level and percentage of accidents for each association.

Predictor Variable 1	Predictor Variable 2	Predictor Variable 3	Predictor Variable 4	Target Variable	Confidence	% Accidents
C1	PC6	WH2		TA6	0.811	7.11
C1	PC6	WH2	PO5	TA6	0.809	5.73
PC6	PA4	P1	E1	TA6	0.807	7.65
PC6	PA4	PO5		TA6	0.806	6.38
S3	PC6	P1	WH2	TA6	0.796	5.68
PC6	WH2	PO5		TA6	0.795	9.23
PC6	DH2	PO5		TA6	0.794	6.04
C1	PC6	PO5	E1	TA6	0.792	10.75
PC6	P3	PO5		TA6	0.792	6.35
C1	PC6	P1		TA6	0.786	7.30
C1	PC6	P1	PO5	TA6	0.785	5.25
PC6	DH3	PO5		TA6	0.783	5.56
PC6	P1	PO5		TA6	0.771	8.38
S3	PC6	PO5		TA6	0.771	5.17
PC4	PA6	P1	PO5	TA2	0.766	6.25
S3	E1	PC6	PO5	TA6	0.753	5.34
C1	PC4	PO5		TA2	0.750	5.68
S3	PC6	PO5		TA6	0.745	6.40
PC6	WH3	PO5		TA6	0.743	5.73
C1	PA6	PO5		TA2	0.464	6.44

**Table 6:** Number of times a variable appears in an association rule for Scenario I.

Variables	PC	C	PO	WH	S	PA	DH	A	E
**Num. of repetitions**	75	57	47	42	39	33	17	15	7
**Ranking**	1	2	3	4	5	6	7	8	9

**Table 7:** Number of times a variable appears in an association rule for Scenario II.

Variables	PO	C	PC	WH	P	DH	PA	E	S	A
**Num. of repetitions**	64	42	37	32	25	20	16	13	10	4
**Ranking**	1	2	3	4	5	6	7	8	9	10

**Table 8:** Data from the Spanish Ministry of Employment and Social Safety between 2005–2015.

Variable	≤49 Workers	50–99 Workers	100–499 Workers	≥500 Workers
**Accidents**	12.2%	9.4%	50.5%	27.9%
**Workers**	4.2%	5.3%	25.8%	64.7%
**Risk Index**	2.9	1.8	2.0	0.4
